# Editorial: Non-reactogenic vaccines against Q-fever

**DOI:** 10.3389/fimmu.2023.1196522

**Published:** 2023-05-05

**Authors:** Anthony E. Gregory, D. Huw Davies

**Affiliations:** Vaccine Research & Development Center, University of California Irvine, Irvine, CA, United States

**Keywords:** *Coxiella burnetii*, reactogenicity, vaccine, hypersensitivity, Q-fever, T cell, DTH, cytokine

Developing safer vaccines against *Coxiella burnetii*, the causative agent of Q-fever, have been challenged with recapitulating the robust efficacy achieved by whole-cell inactivated derivatives. A single dose of formalin inactivated *C. burnetii* delivers a durable immune response that mitigates infection in high-risk populations. Most notably was the significant decline in Q-fever cases in Australia between 2002 and 2006 when Q-Vax was endorsed by the Australian government for the abattoir workforce. However, there are two significant drawbacks to *C. burnetii* whole-cell vaccines. The first is the challenges associated with propagating large volumes of a BSL3 Select Agent for manufacturing. The second is the severe reactogenic response elicited upon immunizing individuals who have been previously exposed to the bacterium. Adverse reactions to this vaccine have included fever, headache, myalgia, and local reactions at the injection site, and in rare cases, more severe reactions such as hepatitis and myocarditis have been reported. In this Research Topic, we provide an update on the continued efforts by researchers working to develop non-reactogenic *Coxiella* vaccines that are both safe and effective.

Fratzke et al. provide a comprehensive review of the current state of Q fever vaccine development and discuss the immunogenicity and reactogenicity of various Q fever vaccine candidates. This paper highlights the challenges associated with *Coxiella* vaccine development, including the need for a vaccine that is effective against multiple strains of the pathogen and can be administered to different age groups. The review also discusses the potential of different vaccine candidates, including whole-cell inactivated or attenuated *C. burnetii* and subunit vaccines.

Sluder et al. then explore the potential of a human T cell-targeted multi-epitope vaccine for Q fever in animal models. The vaccine was designed to target specific T cell epitopes of the pathogen and was tested in mice and guinea pigs. The results showed that the vaccine was able to induce a strong immune response in both animal models, as evidenced by the production of IFN-gamma and IL-2 by T cells. The authors suggest that this vaccine could be a promising candidate for further development for use in humans.

Binette et al. add to the existing literature by highlighting the importance of considering sex differences in the immune response to Q fever vaccination. They investigated sex differences in early-phase delayed-type hypersensitivity (DTH) responses to Q fever vaccination in a murine model. The authors found that female mice had a stronger DTH response than male mice after Q fever vaccination. The authors suggest that sex hormones may play a role in the immune response to Q fever vaccination, and that this should be considered in the development of Q fever vaccines. This could have implications for the development of Q fever vaccines that are tailored to different sexes.

Paul et al. analyzed *ex vivo* whole blood cytokine responses to *C. burnetii* in individuals with natural exposure to the pathogen and in individuals who received Q fever vaccination. The authors found that both natural exposure and vaccination induced a strong cytokine response, with IFN-gamma and IL-10 being the most prominent cytokines produced. They also observed differences in cytokine responses between individuals with and without previous exposure to the pathogen, suggesting that previous exposure may impact the immune response to Q fever vaccination. This research could inform the development of Q fever vaccines that are tailored to individuals with different levels of previous exposure to the pathogen.

While there is still more work to be done in this area, data from this Research topic adds to the growing literature surrounding the development of non-reactogenic *Coxiella* vaccines. Further research in this field has the potential to improve the safety and efficacy of Q fever vaccines and reduce the risk of adverse reactions in individuals who receive them.

## Author contributions

All authors listed have made a substantial, direct and intellectual contribution to the work, and approved it for publication.

